# McKillip Veterinary College

**Published:** 1899-05

**Authors:** 


### McKILLIP VETERINARY COLLEGE.

The third annual commencement exercises of this College were held in the College Auditorium, 1629 Wabash Avenue, Chicago, Ill., on the evening of March 30, 1899. The proceedings were opened by offering of prayer by Rev. E. C. Snyder, after which the baccalaureate address was delivered by Clarence P. Johnson, followed by "Class History," by J. Harry Danforth; "Class Prophecy," by Lawrence W. Bowlus, and "Class Poem," by William J. Patterson. The presentation of diplomas to the graduates was by the President of the College, Dr. M. H. McKillip; presentation of prizes by Prof. J. M. Wright. The valedictory on behalf of the graduating class was delivered by Clyde S. Hess. The following received the degree of D.V.S.: L. Edgar Almony, Lawrence W. Bowlus, Joel E. Cloud, D.V.S.; Thomas Madison Doram, J. H. Danforth, Charles H. Howard, Walter G. Huyett, V.S.; Clyde S. Hess, Fred. E. Jones, Robert Jay, Edward H. Lawley, V.S.; William J. Patterson, Charles Parke, Otto Schukat, John A. Sloan, B.Sc.; George P. Statter, Grant A. Wehr, V.S.; Willard E. Wight, V.S.

